# Progress in Pediatrics

**Published:** 1900-11-17

**Authors:** 


### PROGRESS IN PEDIATRICS.

**The Administration of Anæsthetics.**—In considering this question as applied to children, M'Cartie and Marshall[11] refer first to the preparation of the patient. "No child under eight or ten years of age should be left more than three hours without food, even before a major operation." Milk may remain undigested for five or six hours, so the best thing to give young children is whey, and to older children meat broth. If the operation is to be a short one, a light meal may be given four hours before operation, and some whey or broth three hours later In the administration of the anæsthetic, crying and struggling are, if possible, to be avoided, and with this object the simplest apparatus, such as a piece of lint, ought to be used until the child is asleep. The dosage of the anæsthetic should be at first continuous, and of gradually increasing strength, while narcosis is to be maintained by intermittent administration. As a routine anæsthetic they object to chloroform, which is never free from danger, and prefer A.C.E. mixture, or a combination of 2 parts of ether to 1 of chloroform. In older children nitrous oxide, followed by ether, may be given with advantage, or ether alone from an inhaler if the child is not nervous. Speaking generally, they advise the use of gas, or gas and oxygen, for small operations; and for long operations ether preceded by gas, A.C.E. mixture, or the mixture of ether and chloroform given above. Local anæsthesia is not suitable for operative purposes in children, owing to the fright and struggling involved.

**The Commoner Neuroses of Childhood.**—This is the subject of the Ingleby lectures recently delivered by O. J. Kauffmann,[12] and is taken to include chorea, saltatory or salaam spasm, tetany, night terrors and dreaming, nocturnal enuresis, migraine and allied conditions, and epilepsy. He points out that these affections agree in being commonest in childhood, more frequent in the female sex, and dependent on similar etiological factors. These etiological factors are (1) emotional causes, (2) toxic causes, and (3) hereditary predisposition. The author takes up the various affections enumerated above seriatim, and shows how frequently gastro-intestinal disorders with toxæmia are present. The toxæmia may be induced by an excessive amount of food, unsuitable food, a catarrhal condition of the stomach or small intestine, or constipation. His general principles of treatment are based on these views. The correction of any dietary errors must be attended to. Meat must be given in moderation, or abolished altogether. Light puddings, fish, green vegetables, and potatoes are to be given, the total amount being carefully regulated. The regular action of the bowels is best secured by a single dose of the mixed sulphates of soda and magnesia either the last thing at night or the first thing in the morning. This or some similar medicine ought to be continued for three or four weeks, and then the dose gradually diminished as the regular habit of a morning evacuation becomes established. In addition, intestinal antiseptics may be required; and of these he has obtained most success from sulpho-carbolate of soda and charcoal. Special treatment is also called for in the case of the different neuroses for the special symptoms.

**The Neurotic Form of Enuresis.**—This, according to Amat,[13] has several varieties, and demands distinct treatment. In the first variety the vesicular muscular fibres are too sensitive to distension; and although this is kept in check during the day by the will, it leads to incontinence as soon as the patient drops asleep. The drugs indicated for the relief of this condition are the bromides, chloral, opium, belladonna, and antipyrin. In the second variety there is hyperæsthesia of the vesical and urethral mucous membrane, a condition manifested by the passage of a catheter, and which is best treated by the ammonio-sulphate of copper. In a third variety the vesical sphincter is not irritable, but weak, as shown by the ease with which a catheter passes through the membranous portion of the urethra. For the relief of this condition, strychnine, rhus aromaticus, and more especially electricity, are indicated.

[11] Treatment, April, 1900. [12] The Lancet, June 30 and July 14, 1900
[13] Bull. Gen. de Ther., Nov. 8, 1899, and Ep. B.M.J., Jan. 6, 1900.

